# CircRNA CBL.11 suppresses cell proliferation by sponging miR-6778-5p in colorectal cancer

**DOI:** 10.1186/s12885-019-6017-2

**Published:** 2019-08-22

**Authors:** Hongbin Li, Xiaodong Jin, Bingtao Liu, Pengcheng Zhang, Weiqiang Chen, Qiang Li

**Affiliations:** 1 Institute of Modern Physics, Chinese Academy of Sciences, 509 Nanchang Road, Lanzhou, 730000 Gansu Province China; 2 Key Laboratory of Heavy Ion Radiation Biology and Medicine, Chinese Academy of Sciences, Lanzhou, 730000 China; 3 Key Laboratory of Basic Research on Heavy Ion Radiation Application in Medicine, Lanzhou, 730000 Gansu Province China; 4 University of Chinese Academy of Sciences, Beijing, 100049 China

**Keywords:** Colon cancer cells, Circular RNA CBL.11, MiR-6778-5p, YWHAE

## Abstract

**Background:**

Radiotherapy (RT) is considered an important therapeutic strategy in the fight against colorectal cancer (CRC). However, the existence of some radioresistance factors becomes the main challenge for the RT. Recently, non-coding RNAs (ncRNAs) have shown an important role in modulating cancer cell responses to ionizing radiation (IR). It is therefore of great significance to elucidate the exact mechanisms of ncRNAs in IR-mediated responses to CRC.

**Methods:**

Microarrays were used to identify specific miRNAs that may be altered in response to IR. Bioinformatics, luciferase reporter analyses were used to explore the targets of miR-6778-5p. CircRNA CBL.11 was identified to bind with miR-6778-5p by bioinformatic analysis, AGO2 immunoprecipitation and biotinylated RNA pull-down assay. Functional experiments, including CCK-8 assay, cell colony formation assay and EdU incorporation were conducted to investigate the biological roles of miR-6778-5p and circular RNA CBL.11.

**Results:**

MiR-6778-5p was suppressed in CRC cells after irradiation. Results of functional experiments indicated that miR-6778-5p promoted the proliferation of CRC cells. Luciferase reporter analyses showed that YWHAE was a target of miR-6778-5p, which mediated the function of miR-6778-5p in the proliferation of CRC cells via the p53 pathway. Furthermore, we have noticed that after carbon ion irradiation, circRNA CBL.11 was increased in CRC cells and could function as a competing endogenous RNA (ceRNA) to regulate YWHAE expression by sponging miR-6778-5p, resulting in regulation the proliferation of CRC cells.

**Conclusion:**

CircRNA CBL.11 may play an important role in improving the efficacy of carbon ion RT against CRC.

**Electronic supplementary material:**

The online version of this article (10.1186/s12885-019-6017-2) contains supplementary material, which is available to authorized users.

## Background

Colorectal cancer (CRC) is a frequently diagnosed malignant cancers worldwide and the third leading cause of cancer-related death. Although the therapeutic option of CRC is determined by the appearance of the tumor, the location, stage and many other factors, radiotherapy (RT) is still one of the main choices in the treatment for CRC alone or in combination with surgery and/or chemotherapy. As mentioned in the literatures, preoperative RT is utilized to reduce tumor volume, intraoperative and postoperative RT to prevent recurrence [1, 2]. Unfortunately, resistance of cancer cells to radiation still limits the efficacy of RT. Several factors, for instance, dysregulated radiosensitivity-related gene expression, are known to influence the cellar radiosensitivity [3, 4]. Recently, high linear energy transfer (LET) radiation such as energetic carbon ions provides an option for treatment against CRC, but improving carbon ion RT further remains a research focus. Mounting evidence indicates that noncoding RNAs (ncRNAs), for example, microRNAs (miRNAs) and circular RNAs (circRNAs), play increasingly appreciated roles in the regulatory process of radiation responses [5–7]. However, their biogenesis processes and potential mechanisms for regulating tumor progression in RT, especially using high-LET radiation, remain largely unknown.

Considerable studies highlight the functional association between miRNAs and the development of diseases, including cancer. It has been reported that miRNAs play a regulatory role in CRC progression. By way of illustration, abnormal downregulation of miR-195 was found to be associated with the enhanced proliferation and migration of CRC [8]. The expression of miR-142-3p is highly correlated with poor differentiation and larger tumor size in CRC [9]. In addition, several miRNAs have also shown a role in directly regulating the expression of genes associated with radiosensitivity [10]. For example, miRNAs that regulate DNA repair were considered potential targets for improving RT [11, 12]. Furthermore, some miRNAs whose expression profiles could be altered upon irradiation are involved in the mechanism of ionizing radiation (IR) response [13]. However, it is clear that the causal effects of miRNAs on tumor radiosensitivity and their action mechanisms in ncRNA regulatory networks are still in their infancy.

CircRNAs are a new class of ncRNAs, which are formed through back-splicing of mRNA or long noncoding RNAs (lncRNAs) exons with neither 5′ caps nor 3′ polyadenylated tails and thereby have more stability than their linear types [14]. Therefore, circRNAs can be a valuable diagnostic and therapeutic strategy for cancer treatment. Previous studies have shown that there are differences in the expression of circRNAs under pathological conditions, suggesting that circRNAs may participate in the process of many diseases, including cancer [15, 16]. Further studies revealed that circRNAs could function as molecular sponges for miRNAs and RNA-binding proteins. Recently, it was reported that circRNAs might also play a role in CRC progression. For example, hsa_circ_000984 could act as a ceRNA by sponging miR-106b to promote the proliferation and migration of CRC cell lines [17]. This regulation mechanism among ncRNAs implies that circRNA has an important function in biological and pathological processes [18]. However, the current research on the regulation of circRNAs in cancer RT is still lacking.

In this study, we performed an investigation of miRNA in HCT116 cell line by microarrays, and confirmed that miR-6778-5p was suppressed in CRC cells after carbon ions irradiation. Results of luciferase reporter indicated that miR-6778-5p directly targets YWHAE. Besides, bioinformatic assay demonstrated that circRNA CBL.11 might function as a ceRNA by sponging miR-6778-5p. The purpose of the present research was to elucidate the role and potential mechanism of circRNA CBL.11 in RT using high-LET radiation for CRC via the miR-6778-5p/YWHAE axis. Collectively, our findings reveal novel evidence that, circRNA CBL.11 could function as a ceRNA to regulate the level of YWHAE by sponging miR-6778-5p and exert a regulatory function for the proliferation of CRC cells under carbon-ion exposure. It can thus be suggested that, circRNA CBL.11-miR6778-5p-YWHAE axis plays an important regulatory role in improving the efficacy of carbon ion RT against CRC.

## Methods

### Cells

Human colon epithelial cell line NCM460, human colon cancer cell lines HCT116 and SW620, human colon adenocarcinoma cell line HT29 and human embryonic kidney cell line HEK293T were obtained from Chinese Academy of Sciences Cell Resource Center (Shanghai, China). NCM460, HCT116, HT29 and HEK293T cells were maintained in DMEM media supplemented with 10% fetal bovine serum while L-15 medium with 10% fetal bovine serum was used for SW620 cell culture.

### Irradiation

Irradiations were performed as that described previously [19]. Sham-irradiated groups were taken as a control.

### Microarray analysis

MiRNA expression profiling by microarray analysis were conducted by a commercial service (Oebiotech). Briefly, small RNAs were isolated from HCT116 cell line after radiation, and then labeled with Cyanine-3-CTP. The fragmentation mixtures were hybridized to a Human miRNA Microarray 21.0 (8*60 K, Design ID: 070156). The feature extraction software 10.7.1.1 (Agilent) is used to analyze the scanned images. Raw data were normalized using Genespring 12.0 (Agilent). Student’s t test was used to identify the differential expression of miRNAs.

### RNA preparation and real-time PCR

Total RNA was isolated using Trizol reagent (Invitrogen, USA). In order to verify the circular character, 1 μg of total RNA was incubated 30 min at 37 °C with/without 1 U/μg of RNase R (Epicentre, USA) and 65 °C for 20 min to kill enzyme activity. To quantify the amount of mature miRNA, we used miRNA First Strand cDNA Synthesis (Sangon, China) and U6 as an endogenous control. To quantify the amount of mRNA and circRNA, cDNA was synthesized from 1 μg of RNA by PrimeScript RT Mix reagent (Takara, China) and GAPDH was used as internal control. Real-time PCR was performed using UltraSYBR mixture (Cwbiotech, China). All analyses were performed using the QuantStudio 5 Real-Time PCR System (Thermo Lifetech ABI, USA). The relevant primers are listed in Additional file 2: Table S1 and Additional file 8.

### Plasmid, siRNAs and miRNA mimic and inhibitor

MiR-6778 mimics, inhibitors (Ribobio, China), YWHAE CRISPR activation plasmid (Santa Cruz, USA) and their respective negative control oligonucleotides were transiently transfected using Lipofectamine 3000 (Invitrogen, USA) at a final concentration of 50 nM according to the manufacturer’s instruction. The siRNA sequences were presented in Additional file 2: Table S1.

### Dual-luciferase reporter assay

The 3′ UTR of YWHAE mRNA (NM_006761.4:921-1827) was amplified from cDNA derived from the total RNA of HEK293T cells and subcloned into the pmiR-RB-REPORTTM dual luciferase reporter vector (Ribobio, China). Mutation reporter vector, with a mutation in the 3’UTR complementary to the seed sequence of miR-6778-5p, was generated by PCR. HEK293T cells were co-transfected with 100 ng of the reporter vectors, together with miR-6778-5p or negative controls mimics (50 nM). Cells were harvested 48 h after the transfection, luciferase assays were performed with the Dual-Luciferase reporter Gene Assay Kit (Beyotime, China) according to the manufacturer’s instruction.

### CCK-8 assay

Cells were seeded in 96-well plates (5 × 10^3^/ well) within 100 μL culture medium. The cell viability was examined using CCK-8 Kit (Biosharp, China) according to the manufacturer’s protocols at the indicated periods (24, 36, 48, 72 h). Cells and 10 μL CCK-8 reagents were incubated for 30 min at 37 °C before testing the absorbance at 450 nm was measured with an Epoch microplate Reader (BioTek, USA).

### 5-Ethynyl-20-deoxyuridine (EdU) assay

Cell proliferation was tested by means of EdU assay using Cell-Light EdU DNA Cell Proliferation Kit (RiboBio, China) according to the manufacturer’s protocol [20]. Images were photographed and counted in three randomly selected fields under an FSX100 microscope (Olympus, Japan).

### Clonogenic assay

After transfection and/or irradiation, cells were plated in 6-well plate (5 × 10^2^ /well), cultured for 2 weeks, and the cells were fixed with 10% formaldehyde, stained with 1% crystal violet. Cell survival was analysed by means of the colony formation assay.

### Western blot analysis

Protein extraction was prepared with RIPA buffer supplemented with protease inhibitor. Protein concentration was determined using BCA assay kit (Thermo Scientific, USA). Protein samples were separated by 12% SDS-PAGE, and then transferred onto PVDF membranes (Millipore, USA). The membranes were stained using an ECL chemiluminescent HRP substrate (Bioworld technology, China) according to the manufacturer’s instruction. The images were acquired using GBOX Chemi XRQ System (Syngene, UK) and protein expression levels were quantified with the Image J software. The antibodies used included primary antibodies against YWHAE (Proteintech, China), AGO2 (Proteintech, China), GAPDH (Proteintech, China), ɑ-Tubulin (Proteintech, China), p53 (Abcam, UK), Bcl-2 (Abcam, UK) and Bax (Abcam, UK). The experiment was repeated three times (Additional file 7).

### Oncomine and GSEA assay

We used Oncomine (https://www.oncomine.org/) to analyse mRNA expression levels of YWHAE in colon cancer tissues. The logarithmic transformation and normalized expression values of YWHAE were extracted and analysed on Oncomine. The *P* value < 0.05 was selected as a threshold for reducing the false discovery rate. The expression data downloaded from TCGA data base were divided into two groups (High expression of YWHAE and Low expression of YWHAE) according to the expression of YWHAE by the value of median, and Gene Set Enrichment Analysis (GSEA) was performed using GSEA 2.2.1.

### ceRNA analysis and target prediction

We predicted the circRNA/miRNA interaction using the CircNet database (http://circnet.mbc.nctu.edu.tw/), and we constructed a circRNA-miRNA-gene regulatory networks using the Cytoscape software. The potential miRNA binding sites on circRNAs were predicted through RNA22 v2 and RNAhybrid.

### AGO2 immunoprecipitation

MiR-6778-5p and NC mimic were transfected into HEK293T cells. 48 h after transfection, AGO2 specific antibody was used for AGO2 immunoprecipitation. Briefly, cells were lysed in RIPA containing proteinase inhibitor and RNase inhibitor. The lysate was mixed with antibody-conjugated agarose beads for 4 h at 4 °C. The beads were then washed five times in precooled PBS and the RNA was isolated using Trizol. Real-time PCR was used for assaying the relative expression of circRNA [21].

### Biotinylated RNA pull-down assay

The pull-down analysis with biotinylated RNA was performed according to the manufacturer’s protocol. In brief, biotin-coupled miRNA capture, HCT116 was transfected with 50 μM of biotinylated miR-6778 mimics or nonsense control (NC) using Lipofectamine 3000. The cells were harvested 24 h after transfection, and then the total RNA was isolated using Trizol. A total of 100 μl streptavidin magnetic beads were added to each reaction tube, and the biotin-coupled RNA complex was pulled down at room temperature for 2 h. After elusion, the abundance of circR CBL.11 was evaluated with real-time PCR.

### Statistics

Results were expressed as mean ± SD (standard deviation) based on at least three independent experiments. Comparison between groups was made using the Student’s t-test and the one-way ANOVA were used to evaluate the significance. *p* < 0.05 was considered to be statistically significant.

## Results

### MiR-6778-5p decreased after carbon ion irradiation

**Fig. 1:**
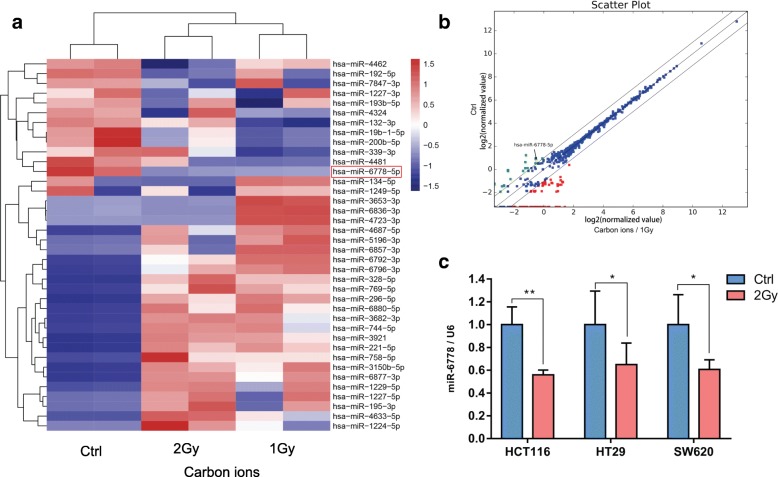
The level of MiR-6778-5p is suppressed in response to carbon ion irradiation. **a** Differential expression of miRNA over 2-fold change and *P* value< 0.05 was analyzed using a cluster analysis. Red indicates a high expression level while green indicates a low expression level. **b** The scatter plot diagram of the chip data was used to evaluate the overall distribution trend of the two sets of the data. **c** MiR-6778-5p level was analyzed using real-time PCR. Data are represented as mean ± SD. *: *P* < 0.05; **: *P* < 0.01 versus the control group

To identify specific miRNAs that may be altered after carbon ion irradiation, we examined the miRNA expression profiles of HCT116 cells before/after different doses of carbon ion irradiation (Additional file 5 and Additional file 6). Under the threshold condition of the change factor ≥ 2.0 and the *P* value < 0.05, 15 miRNAs were up-regulated and 7 were down-regulated (1Gy vs Ctrl); 15 miRNAs were up-regulated and 4 were down-regulated (2Gy vs Ctrl). Differential expression of miRNA over 2-fold change and P value< 0.05 was analyzed using a cluster analysis **(**Fig. 1a**)** and expressed as a scatter plot diagram **(**Fig. 1b**)**. Moreover, real-time PCR was performed to verify the results derived from the microarray. The verification showed that more than 80% of the results are consistent with those of the microarray. Because miR-6778-5p was down-regulated after different doses of radiation treatment, we further validated the data in three different colon cancer cell lines (HCT116, HT29 and SW620). As demonstrated in Fig. 1c, the expression of miR-6778-5p was suppressed in the three different cell lines after carbon ion irradiation, which was observed by real-time PCR.

### MiR-6778-5p promoted the proliferation of CRC cells

**Fig. 2:**
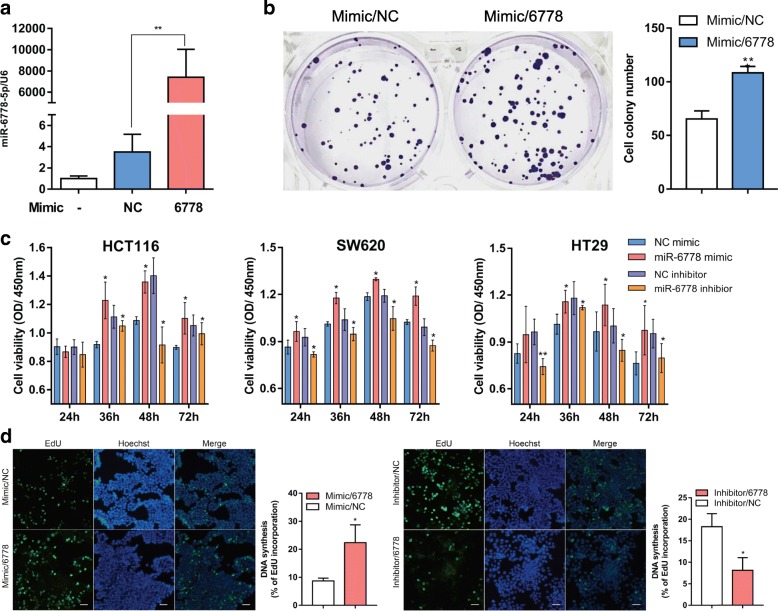
MiR-6778-5p promotes the proliferation of CRC cells. **a** MiR-6778-5p level was analyzed by real-time PCR after transfection with miRNA mimic. **b** Representative image of colony formation capacity. Cell colony number was analyzed from three replicate experiments. **c** Viability of HCT116, HT29 and SW620 cells was measured using CCK-8 assay at 24 h, 36 h, 48 h and 72 h after transfection with miRNA mimic or inhibitor. **d** Analysis of DNA synthesis of HCT116 cells transfected with miR-6778 mimic or inhibitor by EdU assay. Bars, 100 μm. Data are represented as mean ± SD. *P < 0.05, **P < 0.01 versus the control group

We first investigated the role of miR-6778-5p in CRC cells by performing cell colony formation, CCK-8 assay and EdU incorporation assay. Based on the down-regulation in response to carbon ion irradiation, we next assessed the potential functional role of miR-6778-5p by transfecting miRNA mimics to over-express miR-6778-5p (Fig. 2a). After irradiation, forced expression of miR-6778-5p significantly increased the average number of colonies in the colony formation assay compared to control mimic transfected cells (Fig. 2b). The CCK-8 assay also revealed that miR-6778-5p could increase the cell viability remarkably in contrast to the control groups in CRC cells. Conversely, inhibitor of miR-6778-5p significantly suppressed the proliferation of CRC cells (Fig. 2c). In addition, the EdU incorporation assay showed that miR-6778-5p mimic enhanced the proliferation of HCT116 cells, but was impaired by the inhibitory effect of its inhibitor (Fig. 2d). These results indicated that miR-6778-5p might enhance the proliferation of CRC cells.

### YWHAE is the direct target of miR-6778-5p

**Fig. 3:**
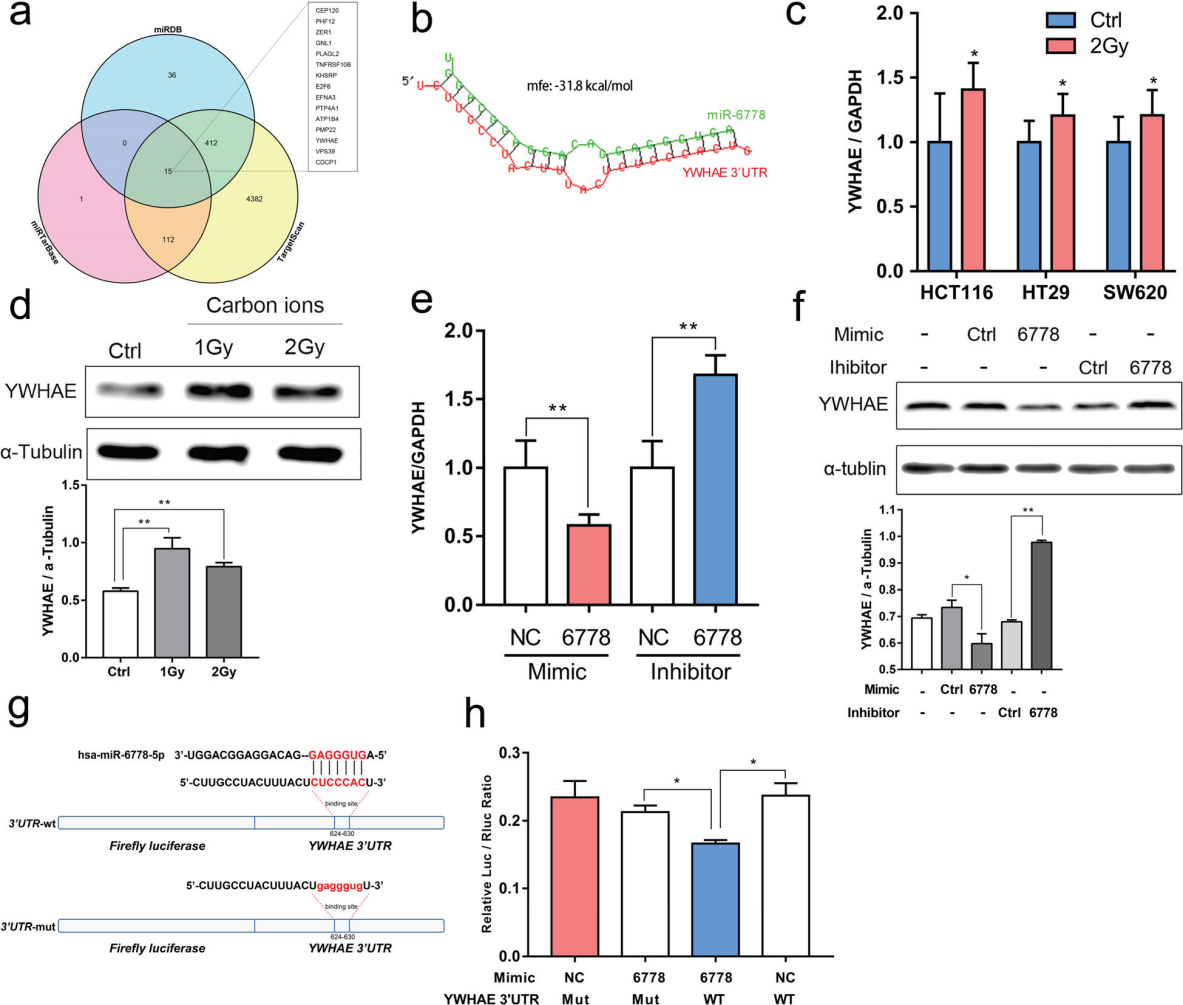
MiR-6778-5p is involved in the regulation of YWHAE expression. **a** A Venn diagram showing the overlap among the predicted targets of miR-6778-5p. **b** Analysis of YWHAE 3′-UTR binding site for miR-6778-5p by RNAhybrid tools. Filtering parameters were as follows: energy threshold<− 20; approximate *p*-value 3utr_human. **c** The levels of YWHAE in CRC cells at 24 h after carbon ion irradiation (2Gy) were measured by real-time PCR. GAPDH was used as a normalized control. **d** The level of YWHAE in HCT116 cells at 48 h after carbon ion irradiation was measured by means of western blot analysis, with α-tubulin serving as the loading control. **e**-**f** HCT116 cells were transfected with a miR-6778-5p mimic (mimic-6778)/ miR-6778-5p inhibitor (inhibitor-6778) or the control mimic (mimic-NC)/ (inhibitor-NC); the expression of YWHAE were detected using real-time PCR and western blot. **g** Dual-Luciferase reporter about YWHAE 3′-UTR binding site for miR-6778-5p. **h** HCT116 cells were co-transfected with miR-6778-5p mimic or control mimic and luciferase reporter containing YWHAE 3′-UTR or its mutant construct. The luciferase activity was detected 24 h after transfection. Data are represented as mean ± SD. *P < 0.05, **P < 0.01 versus the control group

To unravel the molecular mechanism underlying the proliferation promotion of CRC cells by miR-6778-5p, three publicly available databases (miRDB, miRTarBase and TargetScan) were used to predict miR-6778-5p target genes. We identified 13 candidate genes that were commonly predicted to be possible targets of miR-6778-5p from the overlapped part of the three database prediction results. As shown in Fig. 3a, the predicted target genes included ZER1, GNL1, PLAGL2, TNFRSF10B, KHSRP, E2F6; EFNA3; PTP4A1, ATP1B4, PMP22, YWHAE, VPS39 and CDCP1. In addition, the bioinformatics analysis using the RNAhybrid indicated that the 3′-UTR of YWHAE had a potential binding site for miR-6778-5p (Fig. 3b).

Since the expression of miR-6778-5p was down-regulated after radiation treatment, we next investigated the level of YWHAE in cells exposed to carbon ion irradiation by means of western blotting and real-time PCR. Consistent with that expected, the level of YWHAE was up-regulated in CRC cells after carbon ion irradiation (Fig. 3c-d). These results indicated that miR-6778-5p might regulate YWHAE expression both at the mRNA and protein levels. Next, the expression of YWHAE was assessed after transfection of miR-6778-5p mimic or inhibitor in HCT116 cells. The expression of YWHAE was relatively low in the group of miR-6778-5p mimic transfection while it was up-regulated in cells with miR-6778-5p inhibitor transfection (Fig. 3e-f).

To confirm these results, we explored the regulating mechanism using a luciferase assay system. We individually cloned the wild type (WT) and mutated (M) miR-6778-5p recognition elements of 3′UTR-YWHAE to a dual luciferase reporter system (pmiR-RB-REPORT), and tested the ability of miR-6778-5p to regulate the reporters. The results showed that the luciferase activity reduced in cells transfected with the WT YWHAE-reporter. However, the luciferase activity was hardly affected by miR-6778-5p when mutation was introduced into the YWHAE 3′-UTR (Fig. 3g-h). Thus far, the results have argued that YWHAE was a target of miR-6778-5p.

### p53 signaling pathway was regulated by miR-6778-5p via targeting YWHAE

**Fig. 4:**
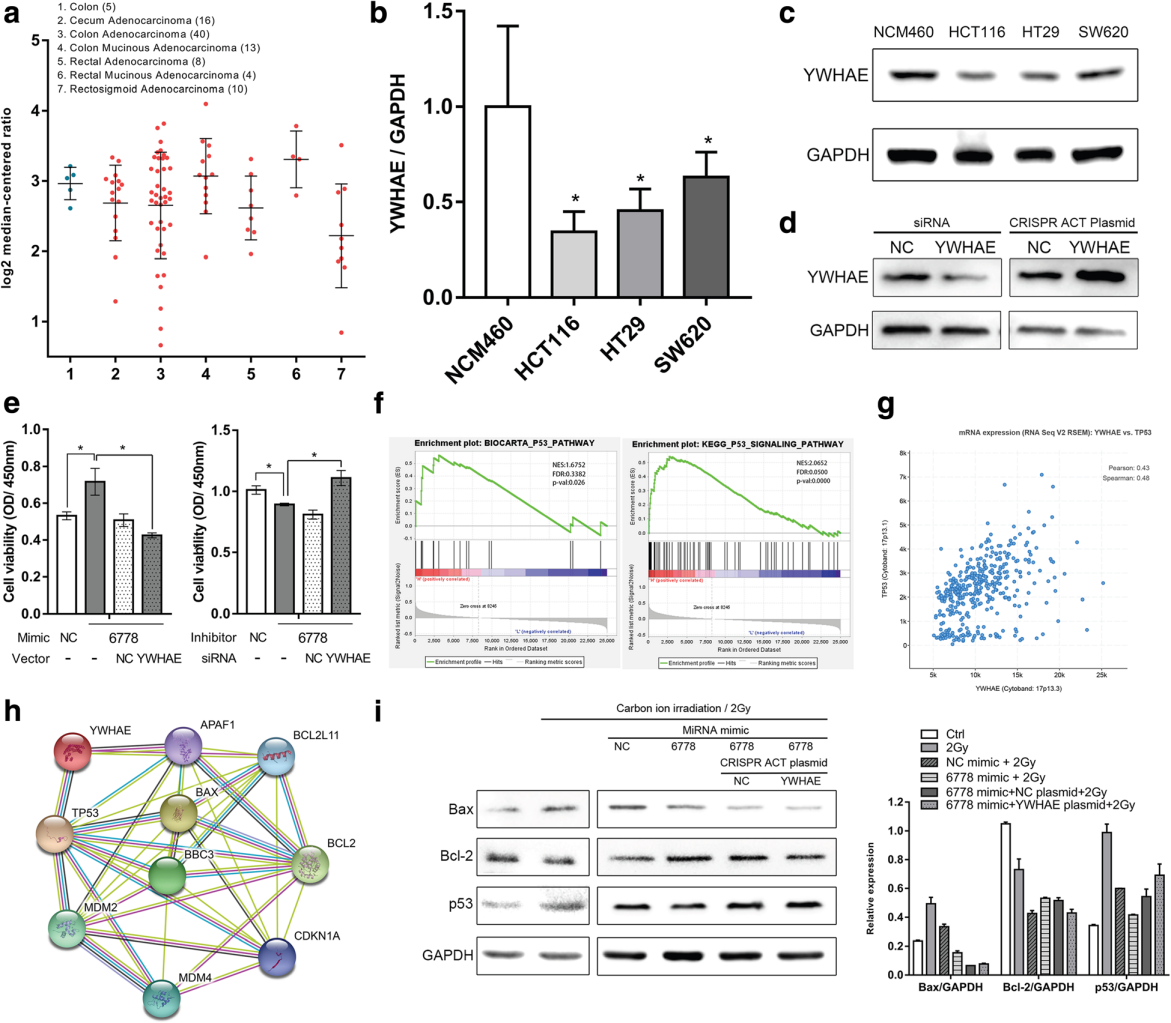
MiR-6778-5p participates in the regulation of cell proliferation by targeting YWHAE. **a** Data mining for YWHAE transcription using the datasets from the Oncomine database. The datasets showed a decreased level of YWHAE mRNA in CRC tissues compared with control tissues. The Y value represents log2 median-centered ratio (normalized expression). **b**-**c** The expression of YWHAE in colon epithelial cells NCM460 and colon cancer cells HCT116, HT29 and SW620 measured by real-time PCR and western blot. **d** The expression of YWHAE in HCT116 cells was measured by western blot after siRNA or CRISPR activation plasmid transfection. GAPDH was used as a control. **e** Viability of HCT116 cells was measured using CCK-8 assay at 24 h after transfection. **f** GSEA enrichment plots indicated that BIOCARTA_P53_PATHWAY and KEGG_P53_PATHWAY gene sets preferentially enriched in YWHAE^high^ samples. NES, normalized enrichment score; FDR, false discovery rate; *P*-Val, nominal *P* value. **g** The expression correlation between YWHAE and p53 in CRC was analyzed by the cBioPortal database. **h** Functional protein association networks of the p53 pathway was analyzed by STRING10.5. **i** The expression of p53, Bax, Bcl-2 in HTC116 cells was measured by western blot under the corresponding treatments. GAPDH was used as a control. Data are represented as mean ± SD. *P < 0.05, **P < 0.01 versus the control group

To further explore the effects of the interaction of miR-6778-5p and YWHAE on CRC cells, we first investigated the level of YWHAE in clinical samples of human colon cancer by performing data mining in publicly available colon cancer datasets using the Oncomine platform (reporter ID: 210317_s_at). Compared with the control group, low YWHAE expression was detected in 62.2% (56/90) of colon cancer tissues examined (Fig. 4a). To confirm this point, we examined its expression with real-time PCR (Fig. 4b) and western blotting (Fig. 4c) in three different CRC cell lines (HCT116, HT29 and SW620) and human normal colonic epithelial cell line (NCM460). We found that YWHAE was expressed at lower levels in CRC cells compared to NCM460 cells. Collectively, the findings above indicated that YWHAE was commonly down-regulated in CRC cells, suggesting that it has a regulatory role in the development of CRC. Given that the role of miR-6778-5p in regulating the proliferation of CRC cells, rescue experiments were conducted by co-transfecting YWHAE CRISPR activation plasmid (Fig. 4d) and miR-6778-5p in HCT116 cells. Then, the CCK-8 assays revealed that over-expression of YWHAE could reverse the proliferation promoting effect of miR-6778-5p. Furthermore, silencing YWHAE by siRNA could also reverse the proliferation repression effect of miR-6778-5p inhibitor (Fig. 4e).

In order to gain insight into YWHAE-mediated molecular pathways in CRC progression, gene set enrichment analysis (GSEA) in the published TCGA colon cancer database (*n* = 521) was performed. The data demonstrated that the expression level of YWHAE was positively correlated with the gene signature of p53 activation (Fig. 4f), indicating that the p53 gene sets may be positively correlated with the expression of YWHAE in CRC cells. Moreover, we analyzed the expression correlation between YWHAE and p53 in CRC by cBioPortal database (http://www.cbioportal.org/). The data showed that YWHAE level was positively correlated with the status of p53. The Pearson and the Spearman coefficient were 0.43 and 0.48, demonstrating their expressions are highly correlated (Fig. 4g). Next, we analyzed the functional protein association networks of the p53 pathway by STRING 10.5 (https://string-db.org/), and found that Bcl-2 associated X proteins (Bax) and B-cell lymphoma-2 (Bcl-2) are major effectors of p53 signaling, in which YWHAE participates. (Fig. 4h). We further set out to determine whether miR-6778-5p could regulate the proteins mentioned above via targeting YWHAE. Western blotting was used to assess the level of p53, Bax and Bcl-2. We have noticed that, after carbon ion irradiation, the level of p53 increased significantly. Meanwhile, compared with the control mimic transfection group, p53 and Bax were suppressed in the miR-6778-5p transfection group; however, the level of Bcl-2 was up-regulated. Next, miR-6778-5p mimic and YWHAE CRISPR activation plasmid were co-transfected into HCT116 cells. The expression trend of the above proteins were reversed by re-introduction of YWHAE post-irradiation (Fig. 4i).

### Circular RNA CBL.11 acted as a competing endogenous RNA for miR-6778-5p

The above results demonstrated that the level of miR-6778-5p was suppressed in CRC cells under radiation stress. This suggested that the up-regulation of certain factors might negatively regulate miR-6778-5p. Given that circRNAs are able to participate in the pathogenesis of various diseases by sponging miRNAs, we next investigated whether circRNAs were involved in regulating miR-6778-5p.

**Fig. 5:**
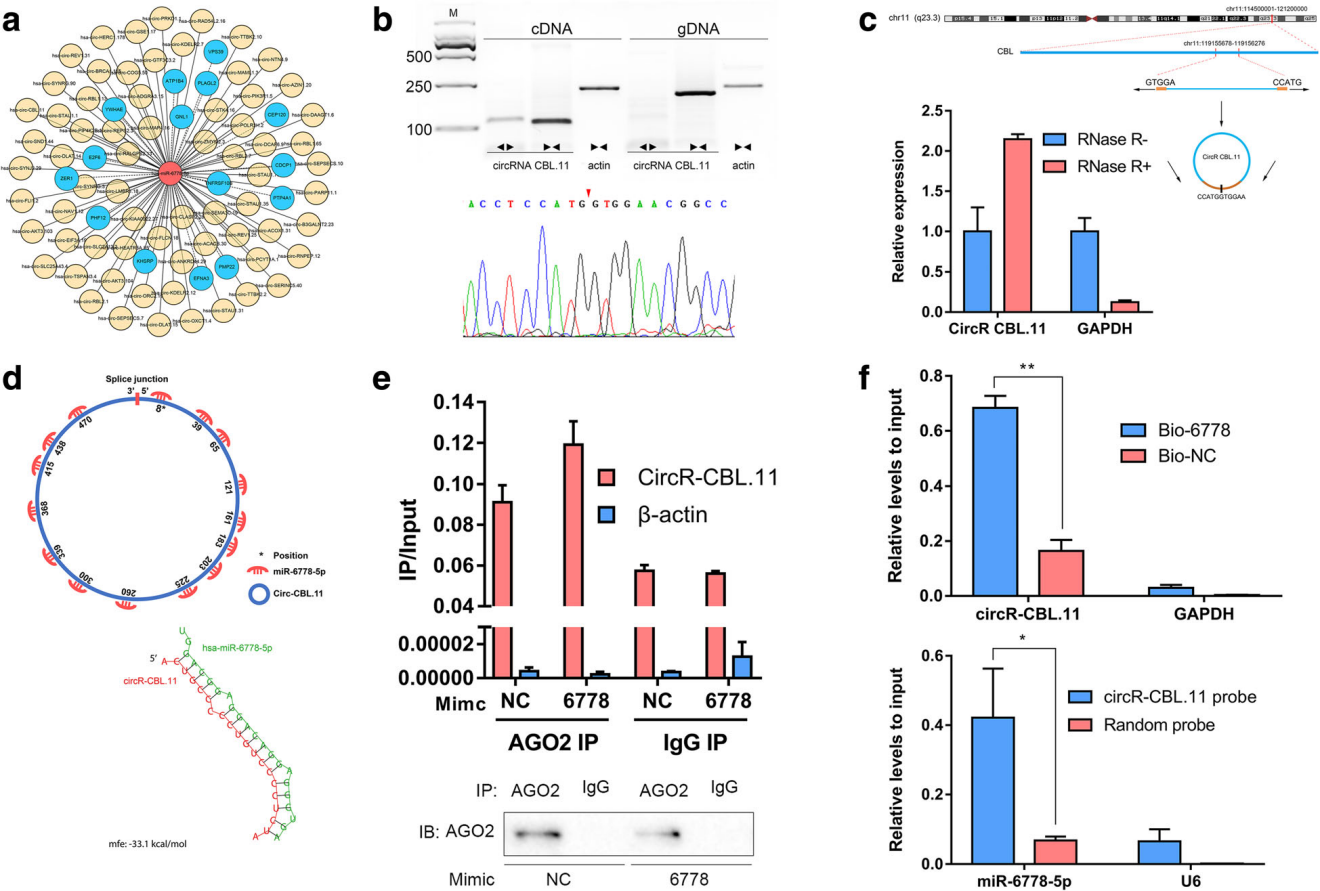
CircRNA CBL.11 functions as a competing endogenous RNA by sponging miR-6778-5p. **a** Cytoscape was used to visualize circRNA-miRNA-mRNA interactions based on the circnet results. In this network, 67 circRNAs (yellow) that interact with miR-6778-5p and the 15 most possible target genes (blue) are presented. **b** Divergent primers amplify circRNA CBL.11 in cDNA but not genomic DNA (gDNA). Sequencing results of the splice junction are shown in the lower panel. **c** Schematic diagram of genomic localization and splicing patterns of circRNA CBL.11. Real-time PCR analysis of circRNA CBL.11 and GAPDH after treatment with RNase R. **d** CircRNA CBL.11 contains 15 binding sites on miR-6778-5p, which was analyzed by RNAhybrid. The binding sites with the smallest minimal free energy are shown in the lower panel. **e** IP of AGO2 from HCT116 cells transfected with miR-6778-5p or the control mimic. The expression of circRNA CBL.11 was detected by qRT-PCR. β-actin was used as a control. **f** The biotinylated miR-6778-5p (Bio-6778) or NC control (Bio-NC) was transfected into HCT116 cells. CircRNA CBL.11 was quantified by real-time PCR, after streptavidin capture. GAPDH was used as a control. In the lower panel, miR-6778-5p was pulled down and enriched with a circRNA CBL.11-specific probe and then detected by rear-time PCR

We first established a circRNA-miRNA-mRNA network using Cytoscape based on the prediction of circnet database (http://circnet.mbc.nctu.edu.tw/) (Fig. 5a**,** Additional file 3: Table S2). Since miR-6778-5p was inhibited after irradiation, we then evaluated the expression pattern of these predicted circRNAs in response to radiation in HCT116 cells using their divergent primers (Fig. 5b). We noticed a substantial increase in circRNA CBL.11, which may be responsible for radiation-induced inhibition of miR-6778-5p. In addition, we analyzed the chromosomal location of circRNA CBL.11 and confirmed its circular character using the RNase R digestion method (Fig. 5c).

Then we characterized the potential miR-6778-5p binding sites on circRNA CBL.11 through RNAhybrid and RNA22 v2. The data showed that, circRNA CBL.11 contains 15 binding sites on miR-6778-5p (Additional file 1: Figure S1). The binding sites with the smallest minimal free energy are shown in the lower panel (Fig. 5d). Subsequently, we applied AGO2 immunoprecipitation in HCT116 cells with miR-6778-5p or control mimic to further confirm this interaction. We extracted RNA from the complex and investigated the level of endogenous circRNA CBL.11 ueing real-time-PCR. As shown in Fig. 5e, circRNA CBL.11 was clearly detectable in the miR-6778-5p transfection group, but was hard to detect in the control mimic transfected cells. These results may indicate that miR-6778-5p facilitated the AGO2 association with circRNA CBL.11.

Next, we conducted a biotin-coupled miRNA capture assay using biotin-coupled miR-6778-5p mimic (Bio-6778) and control mimic (Bio-NC). The data demonstrated that biotin-coupled miR-6778-5p could capture more circRNA CBL.11 than biotin-coupled NC (Fig. 5f), indicating that miR-6778-5p might bind to circRNA CBL.11. Moreover, we performed the biotin-coupled probe pull-down assay to further confirm this interaction (RNA sequences are listed in Additional file 2: Table S1). As expected, a great amount of circRNA CBL.11 and miR-6778-5p was detected in the circRNA CBL.11 pulled down pellet compared with the control group (Fig. 5f), suggesting that circRNA CBL.11 might directly sponge miR-6778-5p. Altogether, these data were consistent with the notion that circRNA CBL.11 could sponge miR-6778-5p and possibly inhibit its activity in CRC cells.

### CircRNA CBL.11 regulated the proliferation of CRC cells through miR-6778-5p and YWHAE

**Fig. 6:**
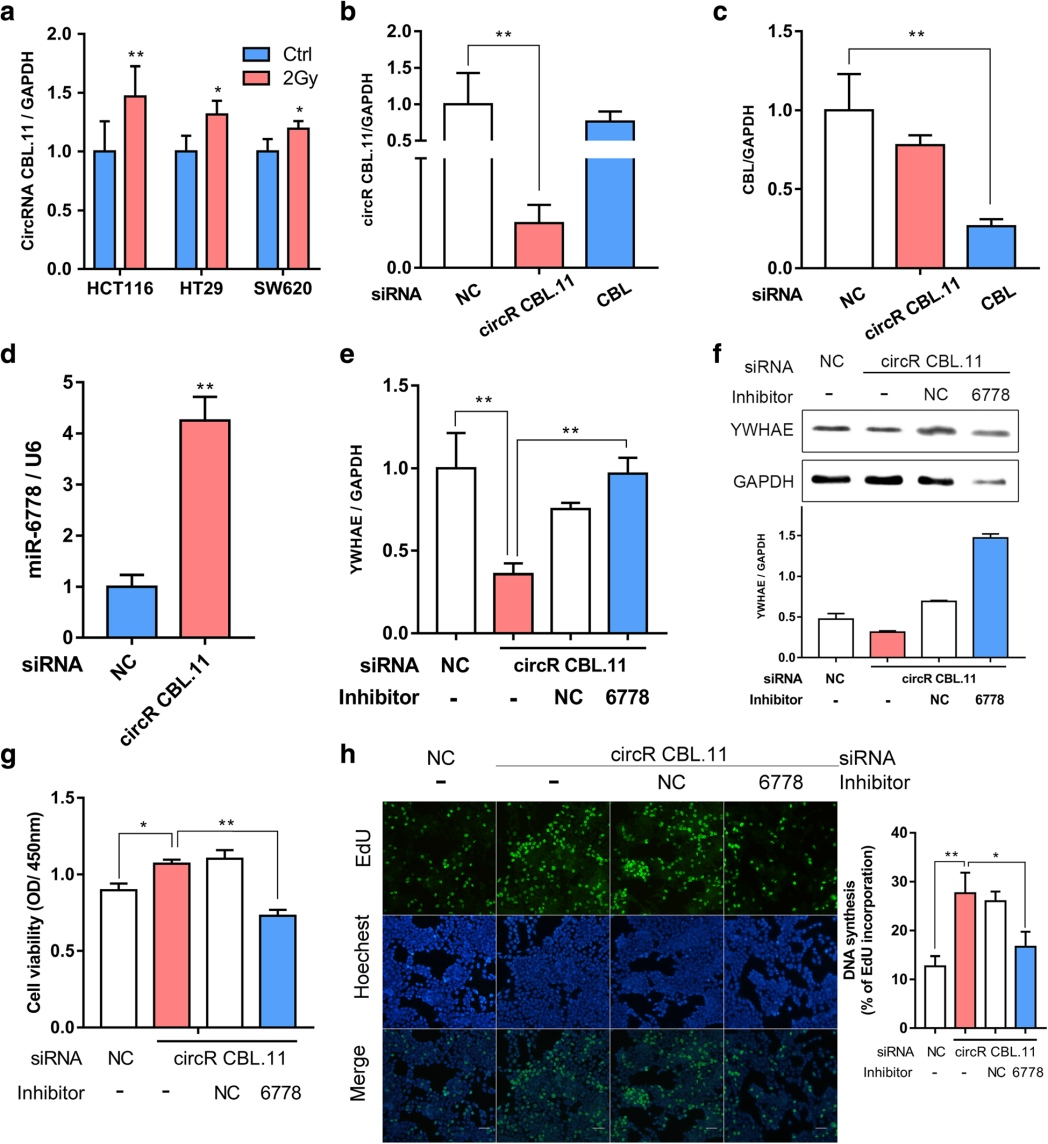
CircRNA CBL.11 regulates the cell proliferation through miR-6778-5p and YWHAE. **a** The levels of YWHAE in CRC cells at 24 h after carbon ion irradiation (2Gy) were measured by real-time PCR. GAPDH was used as a control. **b**-**c** CircRNA CBL.11 and CBL were detected using real-time PCR after siRNA transfection. si-circRNA CBL.11 only knocked down circular transcripts but it is difficult to affect the expression of linear species. However, si-CBL only knocked down its linear transcript, it did not affect the circular transcript. GAPDH was used as a control. **d** MiR-6778-5p level was analyzed by real-time PCR after transfection with si-circRNA CBL.11 and NC siRNA, which was normalized to the U6 expression level. **e**-**f** HCT116 cells were transfected with siRNA and/or inhibitor, and the expression of YWHAE was measured by real-time PCR and western blot. The horizontal line indicates the same processing. **g** Viability of HCT116 cells was measured using CCK-8 assay at 24 h after transfection with siRNA and/or miRNA inhibitor. The horizontal line indicates the same processing. **h** Analysis of DNA synthesis of HCT116 cells transfected with siRNA and/or miRNA inhibitor by EdU assay. Bars, 100 μm. Data are represented as mean ± SD. *P < 0.05, **P < 0.01 versus the control group

Given that circRNA CBL.11 was increased in response to carbon ion irradiation in our study (Fig. 6a), we further investigated its potential functional role by suppressing circRNA CBL.11 in HCT116 cell line. We used circRNA CBL.11 specific siRNAs to knock down its expression and then detected the level of YWHAE in HCT116 cells. A specific siRNA that targeted the back splice junction sequence of circRNA CBL.11 and a siRNA that only targeted its linear type were designed respectively. We observed that the siRNA targeting the back splice junction only knocked down circular transcript without affecting the level of linear species (Fig. 6b). Reversely, the siRNA targeting the linear type could only inhibit the CBL linear transcript rather than the circular transcript (Fig. 6c). Next, we examined the expression of miR-6778-5p and YWHAE after si-circRNA CBL.11 was transferred, and found that the knockdown of circRNA CBL.11 could increase the expression of miR-6778-5p (Fig. 6d), and decrease the expression of YWHAE at both the mRNA (Fig. 6e) and protein levels (Fig. 6f). Furthermore, we co-transfected si-circRNA CBL.11 and miR-6778-5p inhibitor into HCT116 cells, and explored the expression level of YWHAE. The expression of YWHAE was shown to increase in the cells co-transfected with si-circRNA CBL.11 and miR-6778-5p inhibitor, compared with the cells transfected with si-circRNA CBL.11 alone (Fig. 6e-f). Overall, these results indicate that, miR-6778-5p inhibitor could reverse the repression of YWHAE of si-circRNA CBL.11.

Based on the results above, we further tested the involvement of circRNA CBL.11 in proliferation of CRC cells. According to the results of CCK-8 (Fig. 6g) and EdU incorporation assays (Fig. 6h), the silencing of circRNA CBL.11 expression increased the proliferation of HCT116 cells compared to the control group. To confirm that the proliferation regulation of circRNA CBL.11 was dependent on the miR-6778-5p, rescue experiments were performed by co-transfecting si-circRNA CBL.11 and miR-6778-5p inhibitor to address whether the proliferation promotion effect of si-circRNA CBL.11could be reversed by miR-6778-5p inhibitor. The CCK-8 and EdU incorporation assays indicated that the cells co-transfected with si-circRNA CBL.11 and miR-6778-5p inhibitor showed a reduction in proliferation compared with si-circRNA CBL.11-transfected cells (Fig. 6g-h). Taken together, these results demonstrated that circRNA CBL.11 was able to inhibit the proliferation of CRC cells and this regulation was dependent on miR-6778-5p.

## Discussion

Accumulating evidence indicates that ncRNAs have an important role in regulating signaling molecules associated with the progression of cancer, thereby providing new strategies to improve cancer therapy. The potential roles of ncRNAs in CRC progression, however, remained elusive. Here, we demonstrate for the first time that that high-LET radiation induces the increase of circRNA CBL.11, which could function as a sponge for miR-6778-5p to reduce the inhibition of YWHAE. Functionally, circRNA CBL.11 could inhibit the proliferation of CRC cells through acting as a ceRNA of miR-6778-5p to enhance YWHAE expression. Taken as a whole, our study reveals that IR-induced increase of circRNA CBL.11 suppresses the proliferation of CRC cells by sponging miR-6778-5p, which is consistent with our hypothesis indeed.

Many ncRNAs display abnormal expression patterns in cancerous tissues [22]. Moreover, exogenous stressors, such as chemicals and IR, may also be factors influencing the expression of ncRNAs [23]. Thus, modulation of these ncRNAs could be a useful therapeutic strategy to overcome radioresistance and/or chemoresistance in cancer treatment. We anticipate that some ncRNAs that could be altered by IR may be involved in the regulation of radiosensitivity of cancer cells, which is also consistent with some previous reports. For example, miR-95 was found to be up-regulated by IR, which promoted radioresistance in a variety of cancer cells [24]. BANCR (BRAF activated non-coding RNAs) significantly increased after radiation treatment, which could enhance the efficacy of RT [25]. Similarly, as newly-recognized ncRNA, circRNAs have also been shown to be involved in cellular radiation responses [26]. Using microarray analysis, we found that miR-6778-5p decreased after carbon ion irradiation in HCT116 cell line, suggesting that its ectopic expression might be connected with cell survival or radioresistance. Furthermore, we verified this result with real-time PCR in three different CRC cell lines. These results implied that up-regulation of certain factors might negatively regulate the expression of miR-6778-5p. In view of the most commonly reported circRNA function as miRNA sponge that regulates miRNA activity [27, 28], we performed a bioinformatics analysis to predict possible circRNAs that interact with miR-6778-5p, a method that was also mentioned in a previous report [29]. Through experiment, we found that high-LET radiation induced the expression level increase of circRNA CBL.11, functioning as a sponge for miR-6778-5p to reduce the inhibition of YWHAE. However, the exact mechanisms by which circRNA CBL.11 could be regulated by IR remain unclear. CircRNAs are derived from variable cleavage of pre-mRNAs, which are mediated by RNA polymerase II [30]. Miguel et al. reported that, DNA damage induced by IR could downregulate Poll II level [31], which may be one of the causes of IR-regulated circRNA expression. Regulation of circRNAs expression is also dependent on cis-regulatory elements and trans-acting factors, which influence the efficiency of back-splicing [30]. For instance, many RNA binding proteins (RBPs) can regulate the formation of circRNAs [32]. Whether and how IR affects RBPs to regulate the expression of circRNA is still unclear. Yang et al. reported that methylation at the 6 position of adenosine (m6A) in RNA could be induced at the DNA damage sites after irradiation [33]. In addition, modulation of m6A of RNA is thought to also affect the half-life of circRNAs [34]. The mechanisms for circRNA CBL.11 to be regulated by IR need to be experimentally verified in our future work.

YWHAE belongs to the 14–3-3 protein family and is involved in the transduction of signaling pathways by binding to phosphoserine-containing proteins. The reduced YWHAE expression has been described in many cancers [35, 36], suggesting that the regulation of YWHAE is associated with cellular processes related to cancer cell survival and growth. We used the Oncomine database for data mining in CRC datasets, and found that the mRNA level of YWHAE in CRC tissues is lower than that in normal counterparts. In addition, we also observed a significant reduction of YWHAE in CRC cells. Decreased expression of YWHAE in tumors may be associated with its tumor suppressor properties. Moreover, we employed the OncoLnc database (http://www.oncolnc.org/), an interactive tool for exploring survival correlations for downloading clinical data related to YWHAE expression, and noticed that a negative Cox coefficient displayed in COAD set, indicating that the low expression of YWHAE increases the risk of death (Additional file 4: Table S3). In the present study, we validated an increased expression of YWHAE in CRC cells following carbon ion irradiation, which contributed to at least part of the effect of RT using high-LET radiation. Interestingly, similar results were also observed in other cell lines [37, 38].

Mutation and abnormal expression of p53 can be detected in most tumor types. Moreover, p53 is one of the key molecules involved in cellular responses to IR [39]. In general, the therapeutic effect of IR on tumors mainly depends on IR-induced DNA damage. The damage results in activation of DNA repair, which also is the cause of p53 accumulation in response to IR [40]. Causing irreparable damage, which in turn causes tumor cell mitotic catastrophe and death, is the driving force for continuous improvement of RT. For instance, compared with conventional radiations, such as X- and γ-rays, high-LET heavy ions produce more DNA double strand breaks (DSBs) that are even harder to repair [41]. Unrepaired or misreported DSBs could lead to cell death/apoptosis [40], which is mainly mediated by the p53 pathway. Recent studies also show that some ncRNAs have the effect of regulating cellular sensitivity to IR for CRC, depending on the p53 pathway [7, 42]. Our bioinformatics and functional experiments definitely showed that circRNA CBL.11 regulates proliferation of CRC cells via the miR-6778-5p/YWHAE axis, and the p53 signaling pathway. In previous studies, co-expressions and co-localization of YWHAE and p53 were identified by chromosome gene mapping [43]. Interestingly, it was reported that YWHAE could enhance the binding of DNA to p53 via causing p53 dimers to form tetramers [44]. However, the molecular mechanism by which YWHAE interacts with p53 is still unclear, and whether the p53 mutation affects its interaction with YWHAE is an important issue. Therefore, further studies on the molecular mechanism underlying YWHAE involvement in p53-mediated apoptosis-related signaling pathway may offer a greater understanding of radioresistance and/or radiosensitivity.

Mounting evidence shows that circRNAs are not just products of splicing errors, but can regulate alternative splicing or transcription and participate in cancer pathogenesis as miRNA sponge [45]. For example, hsa_circ_0001946 is up-regulated in CRC, which may play a potentially pro-tumorigenic role, and suppress the proliferation of cancer cells by sponging miR-7 [46]. Interestingly, a reduction of circRNA abundance is observed in more reports. Hsa_circ_001988 was verified to be down-regulated in CRC tissues compared to control samples [27, 47]. Bachmayr-Heyda et al. even found that an overall reduction of circRNAs abundance in CRC cell lines and tumor samples compared to the normal mucosa of CRC patients [48]. The cancer cell-type-specific and tissue-specific expression manner of circRNAs has profound effects on the regulation of tumorigenesis and cancer pathways. Moreover, exogenous stressors, such as chemicals and IR, are also influencing factors that cannot be ignored in the expression of circRNA. Yu et al. found that there were 153 differentially-expressed circRNAs in the response of HeLa cells to IR compared with the control group [49]. More than 90 circRNAs were found to be significantly altered in mouse jejuna after irradiation [50]. At the same time, the current study on relationship between circRNA and radioresistance is still in the infancy. Functional studies of circRNA CBL.11 are quite absent as well.

Taken as a whole, the present study confirmed the regulation of circRNA CBL.11 to proliferation of CRC cells exposed to high-LET carbon ions. We first identified that circRNA CBL.11, miR-6778-5p, YWHAE and the p53 pathway together constitute a signaling pathway that regulates apoptosis and proliferation of CRC cells. Although there are several important findings revealed in the present study, we are still far from understanding the molecular mechanisms underlying the ncRNA regulation to radiation responses. This is hampered mainly by the limited insights into the regulatory network of ncRNA and their underlying mechanisms. For example, what is the cause of radiation-induced circRNA changes? Is the regulatory function of circRNA CBL.11 also able to apply to RT against other tumors using high-LET radiation? To address these questions, future work will be required to further elucidate the mechanisms of ncRNA expression regulation by radiation and shed light on how such processes are modulated in reducing radioresistance so as to improve the efficacy of RT.

## Conclusions

In summary, the present study revealed that circRNA CBL.11expression is significantly increased in CRC cells after carbon ion irradiation. Functionally and mechanistically, circRNA CBL.11 regulates YWHAE expression by sponging miR-6778-5p directly to suppress cell proliferation in colorectal cancer. Our findings suggest that circRNA CBL.11-miR6778-5p-YWHAE axis plays an important regulatory role in improving the efficacy of carbon ion RT against CRC.

## Additional files

Additional file 1 : **Figure S1.** The analysis of potential miR-6778-5p binding sites with circular RNA CBL.11. (PDF 509 kb)

Additional file 2 : **Table S1.** Primers and RNA sequences used in this study. (DOCX 15 kb)

Additional file 3 : **Table S2.** CircRNAs interacting with miR-6778-5p predicted by the Circnet database. (DOCX 15 kb)

Additional file 4 : **Table S3.** Cox regression results for YWHAE. (DOCX 16 kb)

Additional file 5 Raw data of the miRNA Microarray used in this study. (RAR 29608 kb)

Additional file 6 Microarray text file parameter description. (PDF 74 kb)

Additional file 7 Raw data of the Western Blotting used in this study. (RAR 9539 kb)

Additional file 8 Raw data of the Real-time PCR used in this study. (RAR 174 kb)

## Data Availability

All data generated or analyzed during this study are included in this published article [and its supplementary information files].

## Abbreviations

ceRNA: Competing endogenous RNA
CRC: Colorectal cancer
DSBs: DNA double strand breaks
GSEA: Gene set enrichment analysis
IR: Ionizing radiation
LET: Linear energy transfer
m6A: Methylation at the 6 position of adenosine in RNA
ncRNAs: Noncoding RNAs
RBPs: RNA binding proteins
RT: Radiotherapy
