# Supplementary material for: CircRNA CBL.11 suppresses cell proliferation by sponging miR-6778-5p in colorectal cancer
Source: BMC Cancer. 2019 Aug 22;19:826. doi: 10.1186/s12885-019-6017-2 (PMC6704711; doi:10.1186/s12885-019-6017-2)
Supplement: Supplementary file 1 — : Figure S1. The analysis of potential miR-6778-5p binding sites with circular RNA CBL.11. (PDF 509 kb) [file 12885_2019_6017_MOESM1_ESM.pdf]

Position: 300

```
target 5'  A     C                 A 3'
            CUGCC CCUGUCC CCU CU
            GACGG GGACAGG GGG GA
```

mfe: -33.1 kcal/mol

Position: 161

```
target 5' A   AU    A      A          A 3'
           ACC  UGCC    GU CCUCCCAC
           UGG  ACGG    CA GGAGGGUG
miRNA  3'             AGGA            A 5'
```

mfe: -30.5 kcal/mol

Position: 260

```
target 5' C         GUACACCAGGCGA       C    G  3'
           CUUGCCUU              CUGUCC UCCA
           GGACGGAG              GACAGG GGGU
miRNA  3' U                             A    GA 5'
```

mfe: -29.5 kcal/mol

Position: 203

```
target 5' C          A A  GG    A 3'
           ACC GCCUCC G CC  CCAU
           UGG CGGAGG C GG  GGUG
miRNA  3'     A      A A  AG    A 5'
```

mfe: -29.2 kcal/mol

Position: 8

```
target 5' G           CCAUUC   AUGGC      A 3'
           GCC GCCUUCU      UCC     CCCAC
           UGG CGGAGGA      AGG     GGGUG
miRNA  3'     A       C         A         A 5'
```

mfe: -28.7 kcal/mol

Position: 65

```
target 5' G     A          GCAGCGAGUAUGUGU    U A 3'
           ACUUG CCUUCUG CC               UCCC C
           UGGAC GGAGGAC GG               AGGG G
miRNA  3'              A                      U A 5'
```

mfe: -27.5 kcal/mol

Position: 121

```
target 5'  A       UAAGG          G   C 3'
            CUGCUUC     CUG CUUCU GCU
            GACGGAG     GAC GGAGG UGA
miRNA  3' UG               A      G     5'
```

mfe: -26.6 kcal/mol

Position: 339

```
target 5'  G         GG    AA       A 3'
            CUGCC CC   CC  UCCC C
            GACGG GG   GG  AGGG G
miRNA  3' UG      A  ACA        U A 5'
```

mfe: -26.2 kcal/mol

Position: 368

```
target 5' U        CCAAGU    A  GA    U   3'
           AUCUGCC       UCC GU  UCCC
           UGGACGG       AGG CA  AGGG
miRNA  3'                   A  GG    UGA 5'
```

mfe: -26.2 kcal/mol

Position: 39

```
target 5'    A       CU          G G 3'
              GCUUCC  UCC CCCG U
              CGGAGG  AGG GGGU A
miRNA  3' UGGA       AC   A    G    5'
```

mfe: -26.0 kcal/mol

Position: 415

```
target 5' A   G  A   A            U 3'
           ACC GC CUC    CUUCCAUU
           UGG CG GAG    GAGGGUGA
miRNA  3'     A      GACAG          5'
```

mfe: -24.7 kcal/mol

Position: 470

```
target 5' U    A G   GGAAGCACGUUCAGU   GAUA    A    3'
           GCCU G CUC               CUG    CCUCC
           UGGA C GAG               GAC    GGAGG
miRNA  3'        G                     A        GUGA 5'
```

mfe: -22.7 kcal/mol

Position: 183

```
target 5'      A    GA A        C 3'
                CUUC  G UCUUCCAC
                GGAG  C GGAGGGUG
miRNA  3' UGGAC    GA A         A 5'
```

mfe: -21.6 kcal/mol

Position: 225

```
target 5' U      GGAGCAGAA   C A    AAAGA   C 3'
           UCUGUU         UCC G CCUC     CGC
           GGACGG         AGG C GGAG     GUG
miRNA  3' U                  A A    G       A 5'
```

mfe: -21.4 kcal/mol

Position: 438

```
target 5'  A   C    ACAAA  GAG     G  3'
            UUG CCUC     UG    CCCA
            GAC GGAG     AC    GGGU
miRNA  3' UG        G      AGGA    GA 5'
```

mfe: -21.0 kcal/mol

Figure S1 The analysis of potential miR-6778-5p binding sites with circular RNA CBL.11.
