# Supplementary material for: CircRNA CBL.11 suppresses cell proliferation by sponging miR-6778-5p in colorectal cancer
Source: BMC Cancer. 2019 Aug 22;19:826. doi: 10.1186/s12885-019-6017-2 (PMC6704711; doi:10.1186/s12885-019-6017-2)
Supplement: Supplementary file 2 — : Table S1. Primers and RNA sequences used in this study. (DOCX 15 kb) [file 12885_2019_6017_MOESM2_ESM.docx]

Table S1 Primers and RNA sequences used in this study

| **Primers for PCR** |  |
| --- | --- |
| CircRNA CBL.11^1^ Forward | GATGTGCCTAGGCTCGGAAG |
| CircRNA CBL.11 Reverse | GGCAGAAGGTCAAGTCGTGG |
| CBL^2^ Forward | TAGGCGAAACCTAACCAAACTG |
| CBL Reverse | AGAGTCCACTTGGAAAGATTCCT |
| YWHAE Forward | GGATACGCTGAGTGAAGAAAGC |
| YWHAE Reverse | TATTCTGCTCTTCACCGTCACC |
| GAPDH Forward | GTCAAGGCTGAGAACGGGAA |
| GAPDH Reverse | AAATGAGCCCCAGCCTTCTC |
| Hsa-miR-6778-5p Forward | Ribobio |
| Hsa-miR-6778-5p Reverse | Ribobio |
| U6 Forward | Ribobio |
| U6 Reverse | Ribobio |
| **Biotinylated probes** |  |
| CircRNA CBL.11 | CGTTCCACCATGGAGGTATC-/3bio/ |
| ChIRP-Probe_NC | TACTGCAGATCGGACTGCCT-/3bio/ |
| (biotin)miR-6778-5p mimics | Ribobio |
| (biotin) NC mimics | Ribobio |
| **siRNAs** |  |
| CircRNA CBL.11 | GGATACCTCCATGGTGGAA |
| YWHAE | CACTTATCATGCAGTTGTT |
| CBL | CTGGACAGGAAGAGAATTA |

1. Alias in circBase: hsa_circ_0095155.

2. linear-CBL (NM_005188.3).
