# Supplementary material for: CircRNA CBL.11 suppresses cell proliferation by sponging miR-6778-5p in colorectal cancer
Source: BMC Cancer. 2019 Aug 22;19:826. doi: 10.1186/s12885-019-6017-2 (PMC6704711; doi:10.1186/s12885-019-6017-2)
Supplement: Supplementary file 3 — : Table S2. CircRNAs interacting with miR-6778-5p predicted by the Circnet database. (DOCX 15 kb) [file 12885_2019_6017_MOESM3_ESM.docx]

Table S2 CircRNAs interacting with miR-6778-5p predicted by the Circnet database

| Name^1^ | Position | Genemic length | Spliced length | Gene symbol |
| --- | --- | --- | --- | --- |
| hsa_circ_0002785 | chr4:22403053-22404422 | 1369 | 249 | GPR125 |
| hsa_circ_0002240 | chr1:243675625-243736350 | 60725 | 658 | AKT3 |
| hsa_circ_0000199 | chr1:243708811-243736350 | 27539 | 555 | AKT3 |
| hsa_circ_0112521 | chr1:235628952-235634274 | 5322 | 190 | B3GALNT2 |
| hsa_circ_0095155 | chr11:119155678-119156276 | 598 | 510 | CBL |
| hsa_circ_0064789 | chr3:33725850-33731424 | 5574 | 370 | CLASP2 |
| hsa_circ_0004350 | chr10:120832401-120833449 | 1048 | 492 | EIF3A |
| hsa_circ_0000370 | chr11:128628009-128651918 | 23909 | 637 | FLI1 |
| hsa_circ_0003948 | chr7:6505701-6509385 | 3684 | 412 | KDELR2 |
| hsa_circ_0005255 | chr3:48019354-48040369 | 21015 | 311 | MAP4 |
| hsa_circ_0002869 | chr1:201681944-201687883 | 5939 | 469 | NAV1 |
| hsa_circ_0006411 | chr5:67522117-67522837 | 720 | 720 | PIK3R1 |
| hsa_circ_0043379 | chr17:36933939-36943173 | 9234 | 648 | PIP4K2B |
| hsa_circ_0060229 | chr20:35668555-35684059 | 15504 | 540 | RBL1 |
| hsa_circ_0069340 | chr4:25156619-25160729 | 4110 | 587 | SEPSECS |
| hsa_circ_0069338 | chr4:25153581-25160729 | 7148 | 690 | SEPSECS |

1. Alias in circBase (http://www.circbase.org/).
