# Supplementary material for: CircRNA CBL.11 suppresses cell proliferation by sponging miR-6778-5p in colorectal cancer
Source: BMC Cancer. 2019 Aug 22;19:826. doi: 10.1186/s12885-019-6017-2 (PMC6704711; doi:10.1186/s12885-019-6017-2)
Supplement: Supplementary file 4 — : Table S3. Cox regression results for YWHAE. (DOCX 16 kb) [file 12885_2019_6017_MOESM4_ESM.docx]

Table S3 Cox regression results for YWHAE

| Cancer^1^ | Cox Coefficient^2^ | P-Value | FDR Corrected | Rank | Median Expression | Mean  Expression |
| --- | --- | --- | --- | --- | --- | --- |
| SARC | 0.313 | 3.10e-03 | 5.69e-02 | 885 | 7662.34 | 8394.75 |
| LUAD | 0.147 | 5.80e-02 | 2.21e-01 | 4398 | 8613.61 | 9341.24 |
| SKCM | 0.138 | 6.00e-02 | 1.92e-01 | 5025 | 10288.77 | 10439.73 |
| HNSC | 0.122 | 8.10e-02 | 3.86e-01 | 3497 | 12320.58 | 12627.09 |
| ESCA | 0.102 | 4.40e-01 | 9.77e-01 | 7561 | 8837.17 | 9308.16 |
| PAAD | 0.095 | 4.30e-01 | 6.33e-01 | 11689 | 7263.72 | 7733.77 |
| BLCA | 0.087 | 2.50e-01 | 5.15e-01 | 7948 | 10919.96 | 11617.24 |
| READ | 0.049 | 8.10e-01 | 9.92e-01 | 13464 | 9964.36 | 10458.9 |
| LUSC | 0.039 | 5.80e-01 | 8.93e-01 | 11028 | 10741.32 | 11956.06 |
| BRCA | -0.019 | 8.30e-01 | 9.40e-01 | 14692 | 10533.47 | 11068.73 |
| OV | -0.021 | 7.90e-01 | 9.64e-01 | 13875 | 11966.51 | 13761.77 |
| GBM | -0.022 | 8.00e-01 | 9.75e-01 | 13812 | 23013.65 | 24619.49 |
| STAD | -0.029 | 7.30e-01 | 9.17e-01 | 13478 | 7655.95 | 8245.83 |
| LAML | -0.031 | 7.60e-01 | 9.20e-01 | 12599 | 5795.2 | 5924.4 |
| LIHC | -0.038 | 6.50e-01 | 8.36e-01 | 12333 | 6757.84 | 7229.0 |
| KIRC | -0.067 | 3.90e-01 | 5.22e-01 | 12467 | 11276.02 | 11597.51 |
| CESC | -0.069 | 6.00e-01 | 8.46e-01 | 11597 | 11818.47 | 11952.98 |
| COAD | -0.153 | 1.50e-01 | 5.58e-01 | 4410 | 10209.5 | 10777.3 |
| UCEC | -0.164 | 1.00e-01 | 9.58e-01 | 1740 | 15688.4 | 16359.62 |
| LGG | -0.268 | 5.60e-03 | 1.49e-02 | 6318 | 21485.19 | 21694.42 |
| KIRP | -0.459 | 5.90e-03 | 3.48e-02 | 2787 | 13446.44 | 13359.43 |

1. Cancer name. SARC: Sarcoma; LUAD: Lung adenocarcinoma; SKCM: Skin Cutaneous Melanoma; HNSC: Head and Neck squamous cell carcinoma; ESCA: Esophageal carcinoma; PAAD: Pancreatic adenocarcinoma; BLCA: Bladder Urothelial Carcinoma; READ: Rectum adenocarcinoma; LUSC: Lung squamous cell carcinoma; BRCA: Breast invasive carcinoma; OV: Ovarian serous cystadenocarcinoma; GBM: Glioblastoma multiforme; STAD: Stomach adenocarcinoma; LAML: Acute Myeloid Leukemia; LIHC: Liver hepatocellular carcinoma; KIRC: Kidney renal clear cell carcinoma; CESC: Cervical squamous cell carcinoma and endocervical adenocarcinoma; COAD: Colon adenocarcinoma; UCEC: Uterine Corpus Endometrial Carcinoma; LGG: Brain Lower Grade Glioma; KIRP: Kidney renal papillary cell carcinoma.

2. A positive Cox coefficient indicates high expression of the gene increasing the risk of death, while a negative Cox coefficient indicates the opposite.
