# Supplementary material for: CircRNA CBL.11 suppresses cell proliferation by sponging miR-6778-5p in colorectal cancer
Source: BMC Cancer. 2019 Aug 22;19:826. doi: 10.1186/s12885-019-6017-2 (PMC6704711; doi:10.1186/s12885-019-6017-2)
Supplement: Supplementary file 6 — Microarray text file parameter description. (PDF 74 kb) [file 12885_2019_6017_MOESM6_ESM.pdf]

Feature results (FEATURES)

The bottom section of the text file gives descriptions of the results for each feature. Results are reported to 9 decimal places in exponential notation for all result files.

FULL Features Table

Table 21 Feature results contained in the FULL output text file (FULL FEATURES table)*

Features (Green)	Features (Red)	Types	Options	Description
FeatureNum		integer		Feature number
Row		integer		Feature location: row
Col		integer		Feature location: column
Accessions		text		Gene accession numbers
Chr_coord		text		Chromosome coordinates of the feature
SubTypeMask		integer		Numeric code defining the subtype of any control feature
SubTypeName		integer		Name of the subtype of any control feature
Start		integer		Indicates the place in the transcript where the probe sequence starts.
Sequence		text		The sequence of bases printed on the array.
ProbeUID		integer		Unique integer for each unique probe in a design

Table 21 Feature results contained in the FULL output text file (FULL FEATURES table)* (continued)

Features (Green)	Features (Red)	Types	Options	Description
ControlType		integer		Feature control type (See “XML Control Type output” on page 204 for definitions.)
			0	Control type none
			1	Positive control
			-1	Negative control
			-20000	Not probe (See Ch. 4 for definition)
			-30000	Ignore (See Ch. 4 for definition)
ProbeName		text		An Agilent-assigned identifier for the probe synthesized on the microarray
GeneName		text		This is an identifier for the gene for which the probe provides expression information. The target sequence identified by the systematic name is normally a representative or consensus sequence for the gene.
SystematicName		text		This is an identifier for the target sequence that the probe was designed to hybridize with. Where possible, a public database identifier is used (e.g., TAIR locus identifier for Arabidopsis). Systematic name is reported ONLY if Gene name and Systematic name are different.
Description		text		Description of gene
PositionX<br>PositionY		float		Found coordinates of the feature centroid in microns

Table 21 Feature results contained in the FULL output text file (FULL FEATURES table)* (continued)

Features (Green)	Features (Red)	Types	Options	Description
LogRatio (base 10)		float		per feature, log of (rProcessedSignal/gProcessedSignal)
				If SURROGATES are turned off, then:
			-4	if DyeNormRedSig <= 0.0 & DyeNormGreenSig > 0.0
			4	if DyeNormRedSig > 0.0 & DyeNormGreenSig <= 0.0
			0	if DyeNormRedSig <= 0.0 & DyeNormGreenSig <= 0.0
LogRatioError		float		If SURROGATES are turned off, then:
			1000	if DyeNormRedSig <= 0.0 OR DyeNormGreenSig <= 0.0
				IF SURROGATES are turned on, then:
				LogRatioError = error of the log ratio calculated according to the error model chosen
PValueLogRatio		float		Significance level of the LogRatio computed for a feature
gSurrogateUsed	rSurrogateUsed	float	Non-zero value	The g(r) surrogate value used
			0	No surrogate value used

Table 21 Feature results contained in the FULL output text file (FULL FEATURES table)* (continued)

Features (Green)	Features (Red)	Types	Options	Description
gIsFound	rIsFound	boolean	1 = IsFound<br>0 = IsNotFound	A boolean used to flag found features. The flag is applied independently in each channel.<br><br>A feature is considered Found if two conditions are true: 1) the difference between the feature signal and the local background signal is more than 1.5 times the local background noise and 2) the spot diameter is at least 0.30 times the nominal spot diameter.
gProcessedSignal	rProcessedSignal	float		The signal left after all the FE processing steps have been completed. In the case of one color, ProcesssedSignal contains the Multiplicatively Detrended BackgroundSubtracted Signal if the detrending is selected and helps. If the detrending does not help, this column will contain the BackgroundSubtractedSignal.
gProcessedSigError	rProcessedSigError	float		The universal or propagated error left after all the processing steps of Feature Extraction have been completed. In the case of one color, ProcessedSignalError has had the Error Model applied and will contain at least the larger of the universal (UEM) error or the propagated error.<br><br>If multiplicative detrending is performed, ProcessedSignalError contains the error propagated from detrending. This is done by dividing the error by the normalized MultDetrendSignal.

Table 21 Feature results contained in the FULL output text file (FULL FEATURES table)* (continued)

Features (Green)	Features (Red)	Types	Options	Description
gNumPixOLHi	rNumPixOLHi	integer		Number of outlier pixels per feature with intensity > upper threshold set via the pixel outlier rejection method. The number is computed independently in each channel. These pixels are omitted from all subsequent calculations.
gNumPixOLLo	rNumPixOLLo	integer		Number of outlier pixels per feature with intensity < lower threshold set via the pixel outlier rejection method. The number is computed independently in each channel. These pixels are omitted from all subsequent calculations.<br><br>NOTE: The pixel outlier method is the ONLY step that removes data in Feature Extraction.
gNumPix	rNumPix	integer		Total number of pixels used to compute feature statistics; i.e. total number of inlier pixels/per spot; same in both channels
gMeanSignal	rMeanSignal	float		Raw mean signal of feature from inlier pixels in green and/or red channel
gMedianSignal	rMedianSignal	float		Raw median signal of feature from inlier pixels in green and/or red channel
gPixSDev	rPixSDev	float		Standard deviation of all inlier pixels per feature; this is computed independently in each channel.
gPixNormIQR	rPixNormIQR	float		The normalized Inter-quartile range of all of the inlier pixels per feature. The range is computed independently in each channel.
gBGNumPix	rBGNumPix	integer		Total number of pixels used to compute local BG statistics per spot; i.e. total number of BG inlier pixels; same in both channels

Table 21 Feature results contained in the FULL output text file (FULL FEATURES table)* (continued)

Features (Green)	Features (Red)	Types	Options	Description
gBGMeanSignal	rBGMeanSignal	float		Mean local background signal (local to corresponding feature) computed per channel (inlier pixels)
gBGMedianSignal	rBGMedianSignal	float		Median local background signal (local to corresponding feature) computed per channel (inlier pixels)
gBGPixSDev	rBGPixSDev	float		Standard deviation of all inlier pixels per local BG of each feature, computed independently in each channel
gBGPixNormIQR	rBGPixNormIQR	float		The normalized Inter-quartile range of all of the inlier pixels per local BG of each feature. The range is computed independently in each channel.
gNumSatPix	rNumSatPix	integer		Total number of saturated pixels per feature, computed per channel
gIsSaturated	rIsSaturated	boolean	1 = Saturated or<br>0 = Not saturated	Boolean flag indicating if a feature is saturated or not. A feature is saturated IF 50% of the pixels in a feature are above the saturation threshold.
gIsLowPMTScaled Up	rIsLowPMTScaled Up	boolean	1 = Low<br>0 = High	Reports if the feature signal value is from the scaled-up low signal image or from the high signal image
PixCorrelation		float		Ratio of estimated feature covariance in RedGreen space to product of feature standard deviation in Red Green space<br><br>The covariance of two features measures their tendency to vary together, i.e., to co-vary. In this case, it is a cumulative quantitation of the tendency of pixels belonging to a particular feature in Red and Green spaces to co-vary.
BGPixCorrelation		float		The same concept as above but in case of background.

Table 21 Feature results contained in the FULL output text file (FULL FEATURES table)* (continued)

Features (Green)	Features (Red)	Types	Options	Description
gIsFeatNonUnifOL	rIsFeatNonUnifOL	boolean	g(r)IsFeatNonUnifOL = 1 indicates Feature is a non-uniformity outlier in g(r)	Boolean flag indicating if a feature is a NonUniformity Outlier or not. A feature is non-uniform if the pixel noise of feature exceeds a threshold established for a "uniform" feature.
gIsBGNonUnifOL	rIsBGNonUnifOL	boolean	g(r)IsBGNonUnifOL = 1 indicates Local background is a non-uniformity outlier in g(r)	The same concept as above but for background.
gIsFeatPopnOL	rIsFeatPopnOL	boolean	g(r)IsFeatPopnOL = 1 indicates Feature is a population outlier in g(r)	Boolean flag indicating if a feature is a Population Outlier or not. Probes with replicate features on a microarray are examined using population statistics.<br><br>A feature is a population outlier if its signal is less than a lower threshold or exceeds an upper threshold determined using a multiplier (1.42) times the interquartile range (i.e., IQR) of the population.
gIsBGPopnOL	rIsBGPopnOL	boolean	g(r)IsBGPopnOL = 1 indicates local background is a population outlier in g(r)	The same concept as above but for background
IsManualFlag		boolean		Boolean to flag features for downstream filtering in third party gene expression software.
gBGSubSignal	rBGSubSignal	float	g(r)BGSubSignal = g(r)MeanSignal - g(r)BGUsed	Background-subtracted signal. To view the values used to calculate this variable using different background signals and settings of spatial detrend and global background adjust, see Table 33 on page 238.

Table 21 Feature results contained in the FULL output text file (FULL FEATURES table)* (continued)

Features (Green)	Features (Red)	Types	Options	Description
gBGSubSigError	rBGSubSigError	float		Propagated standard error as computed on net g(r) background-subtracted signal.<br>For one color, the error model is applied to the background-subtracted signal. This will contain the larger of he universal (UEM) error or the propagated error.
BGSubSigCorrelation		float		Ratio of estimated background-subtracted feature signal covariance in RG space to product of background-subtracted feature standard deviation in RG space
gIsPosAndSignif	rIsPosAndSignif	Boolean	g(r)isPosAndSignif = 1 indicates Feature is positive and significant above background	Boolean flag, established via a 2-sided t-test, indicates if the mean signal of a feature is greater than the corresponding background (selected by user) and if this difference is significant. To view variables used in the t-test, see Table 33 on page 238.
gPValFeatEqBG	rPValFeatEqBG	float		pValue from t-test of significance between g(r)Mean signal and g(r) background (selected by user)
gNumBGUsed	rNumBGUsed	integer		Number of local background regions or features used to calculate the background used for background subtraction on this feature.
gIsWellAboveBG	rIsWellAboveBG	Boolean		Boolean flag indicating if a feature is WellAbove Background or not,<br>feature passes g(r)IsPosAndSignif and additionally the g(r)BGSubSignal is greater than 2.6*g(r)BG_SD. You can change the multiplier 2.6.

Table 21 Feature results contained in the FULL output text file (FULL FEATURES table)* (continued)

Features (Green)	Features (Red)	Types	Options	Description
gBGUsed	rBGUsed	float	g(r)BGSubSignal = g(r)MeanSignal - g(r)BGUsed	Background used to subtract from the MeanSignal; variable also used in t-test. To view the values used to calculate this variable using different background signals and settings of spatial detrend and global background adjust, see Table 33 on page 238.
gBGSDUsed	rBGSDUsed	float		Standard deviation of background used in g(r) channel; variable also used in t-test and surrogate algorithms. To view the values used to calculate this variable using different background signals and settings of spatial detrend and global background adjust, seeTable 33 on page 238.
IsNormalization		boolean	1 = Feature used;<br>0 = Feature not used	A boolean flag which indicates if a feature is used to measure dye bias
gDyeNormSignal	rDyeNormSignal	float		The dye-normalized signal in the indicated channel
gDyeNormError	rDyeNormError	float		The standard error associated with the dye-normalized signal
DyeNormCorrelation		float		Dye-normalized red and green pixel correlation
ErrorModel			0 = Propagated model chosen by you or by software<br>1 = Universal error model chosen by you or by software	Indicates the error model that you chose for Feature Extraction or that the software uses if you have chosen the “Most Conservative” option
xDev		float		A signal-to-noise parameter used to calculate pValue; calculated differently depending on error model chosen

Table 21 Feature results contained in the FULL output text file (FULL FEATURES table)* (continued)

Features (Green)	Features (Red)	Types	Options	Description
gSpatialDetrendIsIn FilteredSet	rSpatialDetrendIsIn FilteredSet	boolean	1 = Feature in filtered set<br>0 = Feature not in filtered set	Set to true for a given feature if it is part of the filtered set used to detrend the background. This feature is considered part of the locally weighted lowest x% of features as defined by the *DetrendLowPassPercentage*.
gSpatialDetrend SurfaceValue	rSpatialDetrend SurfaceValue	float		Value of the smoothed surface calculated by the Spatial detrend algorithm
gIsLowEnoughAdd Detrend	rIsLowEnoughAdd Detrend	boolean		These points are considered to be in the background for the purposes of spatial detrending and multiplicative detrending. If the Boolean value is true for a given point, it will be used in spatial detrending and not in multiplicative detrending (depends on parameters).
SpotExtentX		float		Diameter of the spot (X-axis)
SpotExtentY		float		Diameter of the spot (Y-axis)
gNetSignal	rNetSignal	float		MeanSignal minus DarkOffset
gTotalProbeSignal		float		This signal is the robust average of all the processed green signals for each replicated probe multiplied by the total number of probe replicates, the EffectiveFeature SizeFraction, the Nominal Spot Area and the Weight. For miRNA analyses
gTotalProbeError		float		This error is the robust average of all the processed green signal errors for each replicated probe multiplied by the total number of probe replicates, the EffectiveFeature SizeFraction, the Nominal Spot Area and the Weight. For miRNA analyses

Table 21 Feature results contained in the FULL output text file (FULL FEATURES table)* (continued)

Features (Green)	Features (Red)	Types	Options	Description
gTotalGeneSignal		float		This signal is the sum of the total probe signals in the green channel per gene. For miRNA analyses.
gTotalGeneError		float		This error is the square root of the sum of the squares of the TotalProbeError. For miRNA analyses.
gIsGeneDetected		boolean		Lets you know if the gene was detected on the miRNA microarray.
gMultDetrendSignal	rMultDetrendSignal	float		A surface is fitted through the log of the background-subtracted signal to look for multiplicative gradients. A normalized version of that surface interpolated at each point of the microarray is stored in MultDetrendSignal. The surface is normalized by dividing each point by the overall average of the surface. That average is stored in MultDetrendSurfaceAverage as a statistic. 1-color only
gProcessed Background	rProcessed Background	float		Indicates the Background signal that was selected to be used (Mean or Median).
gProcessedBkng Error	rProcessedBkng Error	float		Indicates the Background error that was selected to be used (PixSD or NormIQR)
IsUsedBGAdjust		boolean	1 = Feature used<br>0 = Feature not used	A Boolean used to flag features used for computation of global BG offset
gInterpolatedNeg CtrlSub	rInterpolatedNeg CtrlSub	float		Value at the polynomial fit of the negative controls.
gIsInNegCtrlRange	rIsInNegCtrlRange	boolean		Set to true for a given feature if its signal intensity is in the negative control range.
gIsUsedInMD	rIsUsedInMD	boolean		Indicates whether this feature was included in the set used to generate the multiplicative detrend surface.
